# An evaluation of genotyping by sequencing (GBS) to map the *Breviaristatum-e* (*ari-e*) locus in cultivated barley

**DOI:** 10.1186/1471-2164-15-104

**Published:** 2014-02-06

**Authors:** Hui Liu, Micha Bayer, Arnis Druka, Joanne R Russell, Christine A Hackett, Jesse Poland, Luke Ramsay, Pete E Hedley, Robbie Waugh

**Affiliations:** 1 Cell and Molecular Sciences, The James Hutton Institute, Invergowrie, Dundee, Scotland DD2 5DA, UK; 2 Biomathematics and Statistics Scotland (BioSS), Invergowrie, Dundee, Scotland DD2 5DA, UK; 3 Hard Winter Wheat Genetics Research Unit, USDA-ARS and Department of Agronomy, Kansas State University, 4011 Throckmorton, Manhattan, KS 66506, USA; 4 Biomedical Sciences Research Complex, University of St Andrews, North Haugh, St Andrews, Scotland KY16 9ST, UK; 5 Division of Plant Sciences, James Hutton Institute, Invergowrie, The University of Dundee. College of Life Sciences, Dundee, Scotland DD2 5DA, UK

**Keywords:** Barley, Dwarfing gene, Genotyping by sequencing, Physical map

## Abstract

**Abstract**

We explored the use of genotyping by sequencing (GBS) on a recombinant inbred line population (GPMx) derived from a cross between the two-rowed barley cultivar ‘Golden Promise’ (*ari-e.GP*/*Vrs1*) and the six-rowed cultivar ‘Morex’ (*Ari-e/vrs1*) to map plant height. We identified three Quantitative Trait Loci (QTL), the first in a region encompassing the spike architecture gene *Vrs1* on chromosome 2H, the second in an uncharacterised centromeric region on chromosome 3H, and the third in a region of chromosome 5H coinciding with the previously described dwarfing gene *Breviaristatum-e* (*Ari-e)*.

**Background**

Barley cultivars in North-western Europe largely contain either of two dwarfing genes; *Denso* on chromosome 3H, a presumed ortholog of the rice green revolution gene *OsSd1,* or *Breviaristatum-e* (*ari-e)* on chromosome 5H. A recessive mutant allele of the latter gene, *ari-e.GP*, was introduced into cultivation via the *cv.* ‘Golden Promise’ that was a favourite of the Scottish malt whisky industry for many years and is still used in agriculture today.

**Results**

Using GBS mapping data and phenotypic measurements we show that *ari-e.GP* maps to a small genetic interval on chromosome 5H and that alternative alleles at a region encompassing *Vrs1* on 2H along with a region on chromosome 3H also influence plant height. The location of *Ari-e* is supported by analysis of near-isogenic lines containing different *ari-e* alleles. We explored use of the GBS to populate the region with sequence contigs from the recently released physically and genetically integrated barley genome sequence assembly as a step towards *Ari-e* gene identification.

**Conclusions**

GBS was an effective and relatively low-cost approach to rapidly construct a genetic map of the GPMx population that was suitable for genetic analysis of row type and height traits, allowing us to precisely position *ari-e.GP* on chromosome 5H. Mapping resolution was lower than we anticipated. We found the GBS data more complex to analyse than other data types but it did directly provide linked SNP markers for subsequent higher resolution genetic analysis.

## Background

Barley (*Hordeum vulgare* L.) is a diploid (2n = 14) economically important cereal crop and genetic model for small grain temperate cereals. Golden Promise (GP) is a two-rowed UK spring barley cultivar, and is currently the most responsive genotype for barley genetic transformation. Also, because of its unique properties, the malt extracted from GP is used to distil a number of signature Single Malt Scotch whiskies such as Macallan and Glengoyne. It is a primary induced gamma-ray mutant derivative of the barley cultivar Maythorpe, and is known to contain a mutation in *Breviaristatum-e* (*Ari-e*). This mutation in *Ari-e* in GP (*ari-e.GP*, also referred to in the literature as GP *erectoides*) causes a semi-dwarfing phenotype that has been used widely in barley cultivar development (especially in Scotland) to shorten straw length and reduce the severity of lodging. GP is also susceptible to several fungal pathogens, has short awns (as well as being dwarf), reduced internode length and shows a measure of tolerance to salt [1]. Genetic analysis has previously located *ari-e.GP* to barley chromosome 5H as a quantitative trait locus (QTL) influencing plant height, and physiological studies have confirmed its relative insensitivity to the addition of exogenous gibberellic acid (GA_3_) [2]. The *Ari-e* gene has not yet been cloned although it was recently mapped as a height QTL using the tools of contemporary biometrical genetics in a complex three-way cross [3].

Over the past two decades, many molecular tools have been developed in barley to enable genetic research [4-9]. The primary focus has been the construction of molecular marker-based genetic linkage maps that can be leveraged for mapping genes of interest and subsequent marker assisted selection in breeding programs. These have been applied to discover, dissect and manipulate genes determining a range of simple and complex traits. Because of their value, accompanied by their increasing use in genetics and breeding, there has been a continual drive to both reduce marker costs and to avoid ascertainment issues [10] while at the same time enhancing flexibility and marker throughput per assay. It is therefore appropriate that new developments in marker technology are both explored and thoroughly evaluated against the current state of the art. Now that next generation sequencing (NGS) technology has been shown to be capable of discovering and genotyping thousands of markers across almost any genome of interest at low cost and in a single step, a current debate is whether sequence-based genotyping methods are ready to replace many of the established and widely used tools such as highly-multiplex Single Nucleotide Polymorphism (SNP) platforms [9].

Available sequence-based genotyping methods generally rely upon the use of restriction enzymes to produce a reduced representation of the non-repetitive (low copy) regions of the genome. Restriction site-associated genomic DNA (RAD) typing is such an approach and has been used in several species for the construction of linkage maps and application in QTL analyses [11]. In barley, a RAD linkage map was recently produced in a double haploid population and used for QTL analysis [12]. Elshire and colleagues [13] subsequently described a similar but more straightforward method of genotyping by sequencing (GBS) which works effectively in 96-well (or higher) plate assays. GBS was originally developed for high-resolution association studies in maize [14] and, like RAD, has been extended to a range of species with complex genomes. A two-enzyme GBS protocol has now been developed that produces a uniform library for sequencing and has been applied to both wheat and barley [15]. This GBS approach has been shown to be suited to genetic analysis of rapeseed, lupin, lettuce, switchgrass, soybean, and maize [16-20].

In this report, our biological objective was to identify at high resolution the genetic location of the *ari-e.GP* semi-dwarfing gene of cultivated barley. However, as a sequence assembly of the barley genome has just been published [21,22], we also wanted to use a sequence-based genetic marker methodology that would in principle allow us to link directly to the genome sequence assemblies and physical map, ultimately as a shortcut to facilitate the identification of the *Ari-e* gene. We therefore chose to explore use of the two-enzyme based GBS method, using digestion of genomic DNA with a six-base methylation sensitive ‘rare-cutter’ and a four-base ‘common cutter’ enzyme. We combined this with the Illumina NGS platform and developed a downstream informatics pipeline to discover co-dominant (SNPs) in an F_11_ single-seed descent mapping population from a Golden Promise (GP) by Morex (Mx) cross. In contrast to GP, Mx is a tall spring six-rowed North American barley variety with desirable malting and brewing characteristics. Most importantly, Mx is the reference cultivar used in the barley genome sequencing efforts. Our genetic analysis using GBS data from the recombinant inbred (RIL) population confirmed the location of *ari-e.GP* on barley chromosome 5H. In the process we discovered 1,949 high-confidence SNPs that we could associate with contigs in the NGS sequence assemblies and physical map.

## Results and discussion

### Golden Promise by Morex population (GPMx) and variation in plant height

**Figure 1:**
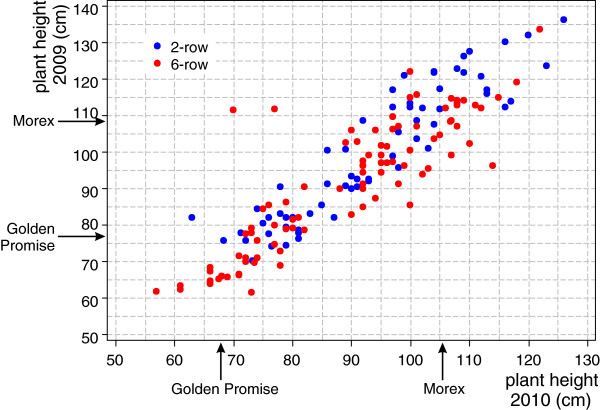
**Scatterplot of plant height measurements of individual GPMx lines collected from the plants grown in polytunnel (in 2009, Y-axis) and in the field (in 2010, X-axis).** Two-rowed lines are shown in blue and six-rowed in red.

A recombinant inbred line (RIL) population of 160 F_11_ single-seed descent lines from a GP by Mx cross was developed over seven years, from 2003–2012, at the James Hutton Institute. The 136 F_11_ RILs used in this study comprised 56 two-rowed and 80 six-rowed accessions. The population segregates quantitatively for height as shown in Figure 1 varying between different RILs from 60 to 130 cm. Asymmetric transgressive segregation of plant height across the GPMx population can be observed – there were around 40 lines taller than Morex, but only about 10 shorter than Golden Promise. The Pearson correlation between the heights in the two years was 0.887. Two lines showed a marked difference in height between the two years.

### Generation of *Pst*I reference sequences from barley genome assemblies

To facilitate genetic analysis by GBS we first extracted a set of 64 bp reference sequences flanking all predicted *Pst*I restriction sites from barley genome assemblies of the cultivars Morex (genome coverage: (53X) [22]), Bowman (26X) and Barke (20X) using the ‘restrict’ program from the EMBOSS suite of tools (see Methods). For cultivar Morex, 343,854 restriction sites were identified, yielding a total of 633,331 GBS reference sequences present on 251,433 unique Morex genome assembly contigs. Of all the identified sites, 54,377 flanking sequences had to be excluded because the restriction site was too close to the start or end of an assembled genomic contig and therefore extraction of the full 64 bp sequence was impossible. Extraction of additional sequences unique to the genome assemblies of cultivars Bowman and Barke yielded a further 71,519 and 97,764 sequences, respectively. After removal of chloroplast (cp) sequences a total of 802,046 reference sequences remained that were subsequently used for read mapping. More than half (54%) of the 64 bp reference sequences stemmed from Morex contigs that contain regions of homology to full length cDNAs or expressed genes, previously mapped genetic markers (cM) or sequences that have chromosome arm assignments based on survey sequencing of flow sorted chromosome arms [23].

### GBS reads of GPMx

We generated three 48-plex GPMx GBS libraries (GPMx_1-3) representing all 136 progenies and the parents, which were repeatedly represented in each library for QC purposes. We used *Pst*I combined with *Mse*I to digest genomic DNA, with the *Pst*I overhang sequence located in the barcode adapter adjacent to the barcode sequence, and the *Mse*I overhang sequence located in common Y-adapter [15] (barcode sequences in Additional file 1: Table S1). Single-end sequencing starting from the barcoded adapter was performed using Illumina chemistry. Pilot sequencing the GPMx_1 library on an Illumina GAII platform generated more than 61 million single-end reads of 72 bp in length. Of these, over 58 M reads were categorised as having a correct barcode and *Pst*I overhang sequence (from here we call this proportion of the sequences ‘categorised reads’). Further sequencing of all three GPMx population libraries, each on one lane of an Illumina HiSeq2000, generated a total of 622 M reads of 100 bp and more than 482 M remaining as categorised reads (see criteria below). The average number of categorised reads obtained per lane was 28.5 M on the Illumina GA II and 160 M on the Illumina HiSeq2000. By applying various filtering criteria (i.e. presence of accurate barcode and complete *Pst*I overhang sequence, and no undetermined nucleotides (Ns) in the reads), the percentages of categorised reads were 93.4% (Illumina GA II) and 77.5% (Illumina Hiseq2000). After deconvolution the distribution of the number of reads per sample ranged from 520,427 to 6,554,933 (Additional file 2: Table S2). The read distribution was relatively even across the population, with only 3 of the 138 lines having less than a million reads. For the parents, we obtained 8.2 M reads from Golden Promise and 11.5 M from Morex, due to repeat sequencing. All sequence reads generated from GPMx were submitted to the Sequence Read Archive section of the European Nucleotide Archive (ENA) (submission: ERP002594 Genotyping by sequencing of a barley mapping population).

### Co-dominant markers from the GPMx population datasets

In total, 461 M categorized reads from the GPMx mapping population were mapped to the 64 bp reference sequences using the Bowtie mapping tool [24]. In order to reduce the number of false positive SNPs during downstream analysis, only a single mismatch per read was allowed, and only uniquely mapped reads were included, leaving 46% of 461 M reads mapped to the reference. These categorized reads were then evaluated for single base-pair differences across the population. We removed all dominant markers from the dataset because of our inability to distinguish null alleles from missing data. Using these highly conservative criteria, we identified an initial set of 1,949 co-dominant SNPs with robust allele calls across the population.

### Linkage mapping of GPMx population

The 1,949 codominant SNPs were analysed using JoinMap. They were first checked for identical pairs based on segregation data across the population and on this basis 267 were excluded, always dropping the SNP marker with the lower quality score in each identical pair. A further 291 SNPs were excluded as they had more than 20% missing values. The remaining 1,391 high confidence SNPs were clustered into seven linkage groups with the number of markers per group ranging from 109 to 270, with nine remaining isolated at a LOD of six. These linkage groups were ordered using JoinMap’s maximum likelihood mapping algorithm. SNPs with a poor fit to the neighbouring SNPs were excluded and the linkage analysis was rerun leaving a total of 1,332 unique high quality SNPs incorporated into the map. The numbers of co-dominant GBS SNPs are presented in Table 1.

**Table 1:** Number of GPMx SNPs mapped

**Chromosome**	**1H**	**2H**	**3H**	**4H**	**5H**	**6H**	**7H**	**Total**
SNPs clustering	151	270	195	109	187	203	267	1,382
SNPs mapped	150	259	176	97	187	201	262	1,332
Additional cosegregating SNPs	20	60	45	12	19	51	57	264

### Location of *SIX ROWED SPIKE 1*

**Figure 2:**
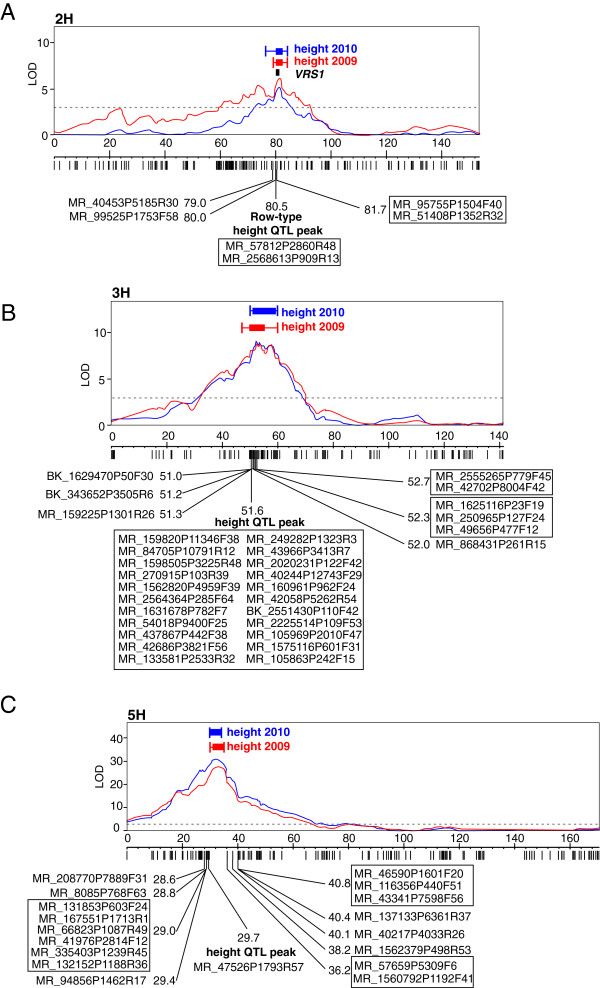
**QTL LOD profiles of plant height and the position of *VRS1*, determined using 138 GPMx RILs.** Only chromosomes with significant QTLs are shown: 2H on panel **A**, 3H on panel **B** and 5H on panel **C**. Vertical lines below the graphs show markers on the GBS map. Marker names and GBS map positions surrounding relevant loci are also shown. Co-segregating markers are outlined. The 2009 data are shown in red and 2010 data are in blue. The horizontal dotted line shows the significance threshold. Boxes show the one-LOD support intervals, and whiskers show the two-LOD intervals.

A major developmental gene, *SIX ROWED SPIKE 1* (*VRS1*), segregates in the GPMx population. The *VRS1* gene has previously been identified [25] and profoundly affects barley spike morphology, but its effect on other plant traits such as height in GPMx is not known. Barley plants carrying a recessive *vrs1* allele (e.g. *vrs1.a* Morex) develop spikes containing six rows of grain in contrast to the ancestral wild type spike which develops only two rows (e.g. *Vrs1.b* Golden Promise). Alternative alleles at *VRS1* also influence the number of tillers that develop on a plant and could, as suggested previously, affect plant height, and this would influence our subsequent analysis of *ari-e.GP.* The most significant associations between row type and SNPs were with MR_2568613P909R13 and MR_57812P2860R48. Both mapped to 80.5 cM on chromosome 2H (Figure 2A). All 80 six-rowed lines had the same genotype as Morex, while the 56 two-rowed lines had the same genotype as Golden Promise (i.e. there were no recombinants between these markers and *VRS1*).

### A major plant height QTL overlaps with the *Breviaristatum-e (Ari-e)* locus

We mapped plant height as a quantitative trait for each year separately using the GPMx GBS linkage map. A permutation test with 1,000 permutations had a 95th percentile of 3.0 for each year’s height data, and this was used as a genome-wide LOD threshold. This resulted in the identification of three significant plant height QTLs on chromosomes 2H, 3H and 5H (Figure 2). The major plant height QTL was located on chromosome 5H (Figure 2C). For each year’s height data, the SNP most closely associated with height was MR_47526P1793R57 at 29.7 cM on chromosome 5H. This SNP explained 55.2% of the variance in height in 2009, with the ‘bb’ genotype having a mean height 27.5 (SE 2.1) cm higher than the ‘aa’ genotype, and 61.6% of the variance in height in 2010, with the ‘bb’ genotype having a mean height 24.8 (SE 1.7) cm higher than the ‘aa’ genotype.

Previously, it was shown that Golden Promise carries a mutation in the dwarfing gene known as *Breviaristratum-e* (*Ari-e*) [26]. The position of the gene has been roughly estimated as about 30 cM from the *SHORT RACHILLA HAIR* (*srh*) locus [27]. Two induced mutant alleles of *Ari-e* (*ari-e.GP* (Golden Promise) and *ari-e.1* (cv. ‘Bonus’)), were introgressed as BC_6_F_3_ lines into the background of cv. ‘Bowman’ resulting in lines BW042 and BW043 [28]. Cross-referencing the SNP markers that define the introgressed region in BW043 (*ari-e.GP*), which has a genetically well-defined introgression, with the barley genome sequence assembly [22] supports *ari-e.GP* as the gene underlying the plant height QTL identified using the GPMx population. This also supports the early observation of *Ari-e* being linked to *srh*[27], as BW873, a nearly isogenic line of cv. Bowman carrying *srh*, contains an introgressed segment located 10–30 cM distal to the GPMx height QTL [28].

Restricted multiple QTL mapping (rMQM) detected two further QTLs for height, the most significant markers being MR_1631678P782F7 at 51.6 cM on chromosome 3H (for both years) (Figure 2B) and a region near *VRS1* (80.5 cM) on 2H (Figure 2A). For the latter, in 2009 the most significant marker was MR_1435185P85F60 at 82.0 cM while in 2010 the most significant marker was MR_48841P1435F22 at 83.2 cM. Regression analysis (in Genstat) was used to model the joint effects of these three locations on height. There were no significant interactions among the three QTLs, and so an additive regression on SNP MR_47526P1793R57 from 5H, SNP MR_1631678P782F7 from 3H and *Vrs1/vrs1* on 2H (for consistency across years) was used. In 2009, these three locations jointly explained 76.6% of the variance in height. For MR_1631678P782F7 on 3H, the ‘bb’ Morex allele has a mean height 9.7 (se 1.7) cm higher than the ‘aa’ GP allele, and the *vrs1* types (six-row) on 2H had a mean height 10.1 (se 1.7) cm lower than the *Vrs1* (two-row) types. In 2010, these three locations jointly explained 77.5% of the variance in height. For MR_1631678P782F7 (3H), the ‘bb’ allele has a mean height 8.9 (se 1.4) cm higher than the ‘aa’ allele and the *vrs1* types (six-row) (2H) have a mean height 6.8 (se 1.4) cm lower than the *Vrs1* (two-row) types. The effect of excluding the two lines with discrepant heights in the two years was investigated, but the QTL locations were unchanged and the differences in the parameter estimates were negligible.

### What is the resolution of the GPMx GBS map at *Ari-e.GP*?

**Figure 3:**
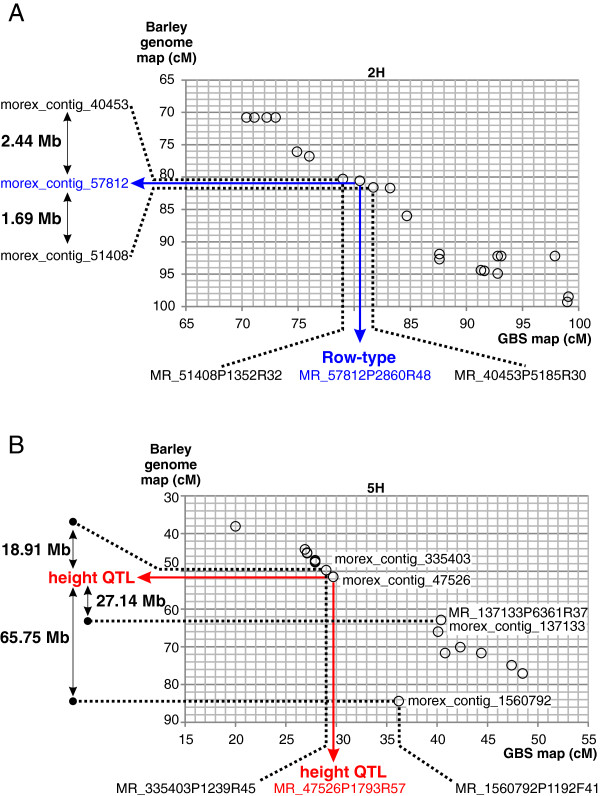
**Scatterplots showing genetic positions of the markers on the GPMx GBS map versus genetic positions of corresponding markers on the barley genome map at the *Vrs1 *(panel A) and *Ari-e *(panel B) loci.** Dotted lines highlight markers that flank these loci on the GPMx map. The map position of *VRS1* and its co-segregating markers are shown in panel **A** in blue. Similarly, the position of the plant height QTL peak and the best marker associated with it is shown on the panel **B** in red. Physical distances between flanking markers and trait loci have been derived from the barley physical map presented in [22].

To explore the potential of using GBS and the GPMx RIL population as a platform for gene identification, we used the barley genome assembly to determine the gene content surrounding *Vrs1*, *Ari-e* and the flanking GBS markers. As the *Vrs1* gene is known [25] we investigated the interval containing the two GBS markers that co-segregated with *Vrs1* and that fell between markers, MR_51408P1352R32 and MR_40453P5185R30. These defined a 1.5 cM interval on the GPMx GBS map. Current information [21,22] indicates this interval corresponds to 4.13 Mb on the barley physical map (Figure 3A). It contains an estimated 52 genes, resulting in a gene density estimate of 12.6 genes/Mb and defining this locus as gene rich (the genome-wide average gene density in barley is 5 genes/Mb).

Unlike *VRS1*, the identity of *Ari-e* is not known. The QTL peak for plant height in GPMx (i.e. the *ari-e.GP* locus) is associated with GBS marker MR_47526P1793R57 which is flanked by MR_335403P1239R45 and MR_1560792P1192F41. These three markers define a 7.2 cM interval on the GPMx GBS map. However on the barley physical map marker MR_137133P6361R37 (morex_contig_137133) appears to be positioned erroneously (Figure 3B) making it difficult to estimate the size of the relevant interval (Figure 3B). Replacing MR_137133P6361R37 with a distal marker, MR_137133P6361R37, the interval defined is 46 Mb and contains an estimated 397 genes. We then investigated a more recent ordering of sequence contigs on the barley genome provided by the POPSEQ methodology [29]. There, the same region corresponds to a 3.3 cM genetic interval in the Barke × Morex population and a 2.1 cM interval in the Oregon Wolfe populations respectively. This region contains over 7,000 anchored sequence contigs spanning a total sequence length of approximately 10 Mb [29]. As the contig sequences represent only a small portion of the physical sequence, *Ari-e* appears to be located in a relatively low recombining region. Despite residual recombination within this genetic interval, the lack of detected polymorphism suggests that the original parental haplotypes may be similar in this region and increased resolution will likely need to be sought in different populations.

## Conclusions

We have shown that GBS is an effective approach for the generation of marker-dense genetic maps in cultivated barley. The short sequence tags enabled us to directly anchor the regions containing both *VRS1* and *ari-e.GP* to the recently released integrated genetic and physical sequence assembly of the barley genome and to crudely define the physical size of the two genetic intervals that we investigated. Our hope was that the flanking markers would ultimately assist us in identifying *ari-e.GP*. Given the resolution we obtained around *ari-e.GP* this seems unlikely. Our data also indicate that a region encompassing the major morphological gene *VRS1,* which determines row-type and number of tillers in barley on 2H and an unknown locus on 3H, also affect plant height in the GPMx population.

An important practical outcome of this work for us was that we found the GBS data more challenging to handle and subsequently to analyse than the current multiplex SNP assay technology we routinely run in the lab [9]. Indeed, this may discourage some groups from adopting the GBS approach. Nevertheless, as the principal determinant of resolution in genetic studies is a combination of the number of recombinants in the population and the number of genetic markers assayed, we were somewhat surprised (and disappointed) that with approximately 1,400 informative genetic markers covering a map length of 1,200 cM, the most closely linked markers to *ari-e.GP* spanned a region of approximately 7 cM. It is possible that the mutant *ari-e.GP* locus was induced within a local haplotype that is shared with Morex (i.e. the gene lies within a region of identity by descent or state between both parents). Indeed, evidence from the barley 9 K iSelect SNP genotyping platform on the parents indicates that GP and Morex probably do share a common haplotype across the proximal short arm and centromeric region of 5H. This would unfortunately result in a markerless gap in the genetic map. Similar gaps have been evident in other high-density genetic maps of barley [9,12,15]. Despite this, the flanking SNP-containing GBS tags will be easy to convert to single-locus markers, and these will be highly valuable for identifying additional recombinants around *ari-e.GP* and, if pursued further using a map based approach, ultimately the identification of the gene.

## Methods

### Plant material and DNA samples

The GPMx population was developed from a cross between a two-rowed barley (Golden Promise, *ari-e.GP*/*Vrs1*) and six-rowed barley (Morex, *Ari-e/vrs1*) at the James Hutton Institute (JHI). DNAs were extracted from one week old seedling tissue using the DNeasy Plant Mini kit (Qiagen). Three 48-plex GBS libraries were constructed from a set of 138 progenies from the F_11_ single-seed descent generation, along with replicated samples of each parent, respectively.

### Plant growth and phenotyping

Ten seeds harvested from a single F_9_ generation plant of the GPMx RIL population were planted in soil in a polytunnel in spring 2009. Planting was randomized and plants grown using automatic watering. Plant height measurements were performed on mature plants prior to harvest. Plant height for each line was determined by selecting the 3–5 longest tillers and measuring the distance from the ground to the top spikelets (excluding awns). Bulked seeds harvested from 3–5 plants of each line of the F_10_ generation of the GPMx RIL population were planted in the field in spring 2010. Before planting, TGW (Thousand Grain Weight) of each sample was determined and used to calculate the weight of the seeds to be planted in 1x 2.5 m plots (so that each plot has about the same number of seeds). In total, 17 randomly selected lines and parents were planted as randomized replicates (2-3X). Plant height measurements were performed 3–4 weeks after anthesis following the same procedure as above. Lodged plants were lifted before measuring their height.

### Constructing GBS libraries

GBS libraries were constructed in a similar manner to Poland *et al*. [15]. Briefly:

A set of 48 barcoded adapters (Additional file 1: Table S1) were generated from complementary oligonucleotides (Sigma) with a *Pst*I overhang sequence and unique barcodes of length 4 nt to 8 nt. In addition, a common Y-adapter was generated corresponding to the 5’ TA overhang generated by *Mse*I. Top and bottom strand complementary oligonucleotides for each adapter (50 μM) were annealed using the following program: 95°C for 2 min, decrease to 25°C by 0.1°C/s, hold at 25°C for 30 min. Annealed adapters were diluted 1:10 and their concentration measured using PicoGreen. Barcoded adapters were normalised to 2 ng/μl and the common Y-adapter to 40 ng/μl.

DNAs were digested in 30 μl reactions containing 200 ng of genomic DNA, 1 × NEB buffer 4, 8 u *Pst*I-HF, 8 u *Mse*I, incubated at 37°C for 3 h, then 80°C for 20 min to inactivate the enzymes. For ligation, 4 ng annealed barcoded adapter and 200 ng annealed common Y-adapter were added along with 1 × T4 DNA ligase buffer and 200 u T4 ligase in a total volume of 50 μl. All 48 ligation reactions were incubated at 22°C for 2 h, then 65°C for 20 min.

An aliquot (5 μl) was removed from each ligation reaction, pooled, purified using QIAquick PCR Purification Kit (Qiagen) and eluted in 30 μl of dH_2_O. PCR amplification was conducted in 50 μl reactions containing 4 μl of pooled and purified library DNA, 1 × high fidelity Phusion polymerase buffer, 0.2 μM dNTP, 0.2 μM primer 1 (complementary to barcode adapter), 0.2 μM primer 2 (complementary to common Y-adapter), 1 u Phusion polymerase Taq. PCR was conducted as follows: 98°C for 30 s for one cycle; 20 cycles of 98°C for 10 s, 65°C for 20 s, 68°C for 20 s; one cycle of 75°C for 5 min, cool to 4°C. The PCR enriched library was gel-purified, selecting the 200–500 bp size fraction, using the MinElute Gel Extraction Kit (Qiagen), eluted in 12 μl dH2O, and quality and quantity of the library measured using a Nanodrop and Agilent Bioanalyzer.

### Sequencing and processing raw GBS data

Single-end sequencing from the *Pst*I sites was carried out using Illumina GA II and/or HiSeq2000 sequencer: of the three GBS libraries (GPMx_1, GpMx_2 & GPMx_3), initially GPMx_1 was sequenced on two lanes of Illumina GAII and subsequently all three GBS libraries were sequenced on one lane each of Illumina HiSeq2000. All GBS sequences were submitted to Sequence Read Archive section of the European Nucleotide Archive (ENA) (submission: ERP002594 Genotyping by sequencing of a barley mapping population).

### Generation of reference sequences

Reference sequences for the mapping of GBS tags were generated from existing genomic assemblies of the barley cultivars Morex, Bowman and Barke based on Illumina whole genome shotgun sequencing. As a first step in the workflow (see Additional file 3: Figure S1 for a diagram of the full workflow), the EMBOSS program *restrict* (http://emboss.sourceforge.net/) was used to discover *Pst*I restriction sites in the assemblies. Custom written Java code was then used to extract from the Morex genomic assembly two separate flanking 64 bp sequences extending the restriction site in forward and in reverse direction. This process was repeated for the other two cultivar assemblies and the extracted 64 bp sequences were then compared with the sequences generated from cultivar Morex assembly using the standalone BLASTN program [30] from NCBI (version 2.2.26+). A single hit was obtained per query, and from this we extracted those hits with alignments along the full length of the query sequence, an identity value of less than 100%, and a mismatch number of at least 2. These hits were added to the full set of Morex flanking sequences, thereby providing a global set of reference sequences from the three barley genome assemblies. To further refine the reference sequences, we screened them for chloroplast DNA, which can be a common feature in whole genome shotgun sequencing. This was done by BLASTN, with the combined set of sequences as query against the full barley chloroplast genome sequence (http://www.ncbi.nlm.nih.gov/nuccore/118430366?report=fasta). Hits were filtered to require sequence identity > = 90%, and an alignment length > = 64. We detected 568 chloroplast DNA sequences that were subsequently removed from the reference set.

### Read mapping

Prior to mapping, the raw Illumina reads were assigned to their respective samples (‘deconvoluted’) based on the sample-specific barcodes included in the sequence. Barcode lengths varied between 4 and 8 bases therefore custom written Java code was used for deconvolution, and this also removed the barcodes after assigning the read to a sample, which is a requirement for the successful mapping of the read to a reference sequence. Reads that started with the *Pst*I overhang sequence (TGCAG) after barcode removal were accepted, quality trimmed to remove bases of quality Phred < 20 from the 3’-end (distal to the *Pst*I site), and then shortened from the 3’-end to a standard length of 64 bases. Reads that were shorter than this after quality trimming were discarded.

Reads were then mapped to the 64 bp reference sequences using the Bowtie mapping tool (version 0.12.7, [24]). To avoid cross-mapping of reads between similar sequences, the “ --best --strata” switch was used, which ensures that multi-mapped reads are only mapped to the location with the fewest mismatches. In order to reduce the number of false positive SNPs during downstream analysis, only a single mismatch per read was allowed (“-v 1”), and only uniquely mapped reads were retained (“-m 1”).

### SNP discovery and genotype calling

We used the FreeBayes software [31] to discover single nucleotide polymorphisms (SNPs), as well as custom Java code for converting the resulting VCF file into a human-readable text file. Within FreeBayes, the SNPs were filtered to retain those where the minimum number of reads with the alternative allele was greater than 3, which provided a total of 57,328 SNPs. We then applied the following filters: the minimum fraction of reads with the alternative allele for a SNP should be greater than or equal to 0.1; the percentage difference between the base qualities for the reference and alternative alleles should be less than or equal to 5; the SNP quality score cut-off should be greater than or equal to 20. This procedure yielded 18,251 SNPs. Then, within Excel, further filters were applied: we required a total read coverage of greater than or equal to 700 (ie. a mean of at least 5 reads for each sample in the population), which left 3,246 SNPs; the percentage of heterozygous samples was less than or equal to 2%, which left 1,985 SNPs; the ratio of alternative allele/reference allele was greater than or equal to 0.5, which left 1,968 SNPs.

Genotypes were then called based on the proportion of the reference allele. We identified this as homozygous for the reference allele if the proportion was greater than 0.8, as homozygous for the alternative allele if the proportion was less than 0.2 and as heterozygous if the proportion is between 0.2 and 0.8. Samples with fewer than three reads if designated homozygous, or with fewer than six reads if designated heterozygous, were recoded as missing. Nineteen SNPs had a missing genotype for one of the parents, and these were also excluded to leave 1,949 SNPs for linkage mapping. Visual inspection of both mappings and SNPs was carried out using the Tablet software [32].

### Linkage mapping

The SNP data were sorted by decreasing quality score before analysis with JoinMap [33]. This ensured that when co-segregating SNPs were excluded, the lower quality SNPs were preferentially dropped. SNPs with greater than 20% missing values were also excluded from the JoinMap analysis. SNPs were grouped using the independence LOD score, and then ordered within each linkage group using the maximum likelihood algorithm. The GBS tags were mapped to reference sequences generated from Morex, Bowman and Barke WGS shotgun assemblies. Those from Morex contain previously published anchored genetic/physical markers, which we assumed to be correct. We define these as anchoring markers on the genetic linkage groups. Additional file 4: Table S3 provides a list of 1,332 unique co-dominant GBS markers used for map construction and ordered according to their map location on the GPMx population. It highlights 403 genetically redundant markers, the correspondence of all GBS tags to expressed genes (MLOC’s) and their genetic position on the IBSC consensus map (IBSC, 2012).

### QTL mapping

QTL interval mapping was used to locate QTLs for the 2009 and 2010 height data separately, using MapQTL [34]. A permutation test with 1,000 permutations was used to establish the LOD threshold. Restricted multiple QTL mapping (rMQM mapping) was used to search for further QTLs taking into account the most significant ones. A regression analysis, using Genstat 15 for Windows [35], was used to test for significant interactions among the selected QTLs. Genstat was also used to test which of the mapped SNPs showed the greatest association with the two-rowed/six-rowed type, using chi-square tests of independence.

### Cross-referencing barley genome data sets

In total 4,607 individual sequences from the manifest files accompanying barley OPA SNP mapping platforms [36] were used to identify corresponding sequences in the barley genome represented by ~2.6 million sequence contigs using the blastN algorithm [9]. The resulting table cross-referenced SNP markers used to define introgressions in the Bowman backcross derived lines [28] and sequence contigs in the barley genome assembly [22]. Tables containing the contig anchoring results from POPSEQ [29] are available for download from ftp://ftp.ipk-gatersleben.de/barley-popseq/.

## Competing interests

The authors declare that they have no competing interests.

## Authors’ contributions

HL, PH, AD and RW conceived the study. HL and PH established and conducted the GBS analysis of the GPMx population with guidance from JP. AD developed and phenotyped the GPMx population. HL, JR, AD, LR and CH conducted statistical analysis of the genetic data with all NGS data processing steps conducted by MB and HL. HL, AD, PH, MB, CH and RW wrote the manuscript, and all authors read and approved the final version. RW and PH supervised the work and obtained the funding that allowed the study to be conducted.

## Supplementary Material

Additional file 1: Table S1 Barcode adapters used for GBS. Click here for file

Additional file 2: Table S2 GBS read distribution. Click here for file

Additional file 3: Figure S1 Data processing workflow for the raw GBS Illumina reads. Click here for file

Additional file 4: Table S3 Co-dominant GBS markers. Click here for file
