# Supplementary figures and images for: An evaluation of genotyping by sequencing (GBS) to map the Breviaristatum-e (ari-e) locus in cultivated barley
Source: BMC Genomics. 2014 Feb 6;15:104. doi: 10.1186/1471-2164-15-104 (PMC3922333; doi:10.1186/1471-2164-15-104)

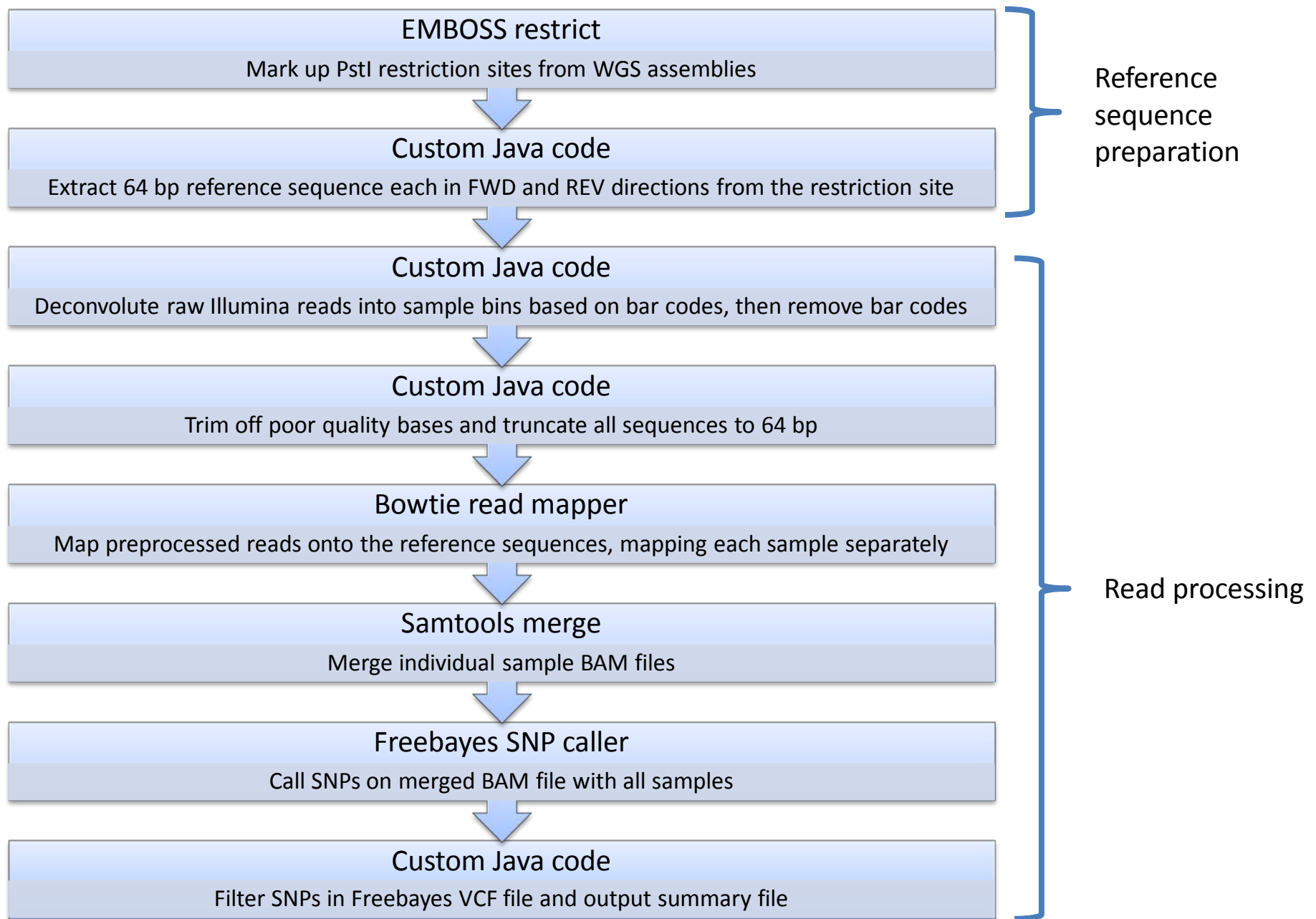

Supplementary Figure 1. Data processing workflow for the raw GBS Illumina reads.

Supplement: Additional file 3: Figure S1 — Data processing workflow for the raw GBS Illumina reads. [file 1471-2164-15-104-S3.pdf]
